# Percutaneous Pharmaco-Mechanical Thrombectomy of Acute Symptomatic Superior Mesenteric Vein Thrombosis

**DOI:** 10.1007/s00270-019-02354-y

**Published:** 2019-10-24

**Authors:** Paolo Rabuffi, Simone Vagnarelli, Antonio Bruni, Gabriele Antonuccio, Cesare Ambrogi

**Affiliations:** Department of Interventional Radiology, “San Giovanni Addolorata” Hospital, Via dell’Amba Aradam 9, 00184 Rome, Italy

**Keywords:** Acute superior mesenteric vein thrombosis, Mesenteric venous ischemia, Percutaneous mechanical thrombectomy, Pharmaco-mechanical thrombectomy, Transcatheter thrombolysis, Aspirex

## Abstract

**Purpose:**

To evaluate the safety and the efficacy of percutaneous pharmaco-mechanical thrombectomy (PPMT) of acute superior mesenteric vein (SMV) thrombosis.

**Methods:**

A database of patients treated between 2011 and 2018 with acute venous mesenteric ischemia (VMI) was reviewed. VMI was diagnosed in the presence of SMV thrombosis and CT evidence of jejunal thickening. All patients presented with mild to moderate peritonism, which allowed surgery to be postponed. Initial treatment consisted of heparinization. PPMT was indicated in case of worsening abdominal pain despite anticoagulation and was performed via a transjugular or transhepatic approach, using a rotational aspiration thrombectomy catheter, followed by transcatheter thrombolysis. Clinical success was defined as symptoms resolution. Technical success was defined as patency of > 50% of SMV at venography and resolution of jejunal thickening. Patients were discharged on lifelong oral anticoagulation (INR 2.5–3.5). Follow-ups were performed using CT and color Doppler ultrasound.

**Results:**

Population consisted of eight males, aged 37–81 (mean 56.5 years). Causes for thrombosis were investigated. Urokinase infusion time ranged from 48 to 72 h (3,840,000–5,760,000 IU). Clinical and technical success was obtained in all cases. One patient experienced bleeding from the superior epigastric artery and was treated with embolization. One patient died of multi-organ failure after 35 days, despite resolution of SMV thrombosis. In no case was surgery required after PPMT; mean hospitalization was 14.1 days (9–24). Mean follow-up of remaining seven patients was 37.7 months (12–84 months).

**Conclusion:**

PPMT of acute SMV thrombosis seems safe and effective, with an 87.5% long-term survival rate and a 12.5% major complication rate.

## Introduction

Acute SMV thrombosis is a rare disease that accounts for 5–15% of mesenteric thromboembolic events [1]. Owing to a delay in diagnosis determined by its vague symptomatological profile, it is associated with high rates of mortality, up to 50% in the case of intestinal infarction [2]. The causes of acute SMV thrombosis include liver cirrhosis, a previous history of surgery, abdominal infection or inflammation, and hypercoagulable states (such as deficiencies of antithrombin III, protein C, protein S, and factor V Leiden) [3]. Systemic anticoagulation with heparin is the initial conventional therapy for acute SMV thrombosis. Surgery is indicated in the presence of clinically severe peritonism, or in the case of bowel infarction or perforation at CT, but it is burdened with high mortality (up to 39%) and morbidity (32–71%) rates [4]. In recent years, endovascular treatment options such as transcatheter thrombolysis and percutaneous mechanical thrombectomy have been increasingly used in cases of acute SMV thrombosis in order to achieve early revascularization [5] and have been shown to lower mortality rates, reduce hospitalization stays, and diminish the impact of surgical resection [6].

The aim of this retrospective series is to investigate the safety and efficacy of percutaneous pharmaco-mechanical thrombectomy in the treatment of symptomatic acute SMV thrombosis with the use of the Aspirex thrombectomy device (Straub Medical, Wangs, Switzerland), followed by local thrombolysis.

## Methods

The database of patients admitted to our department for acute abdominal pain (symptom onset < 14 days), caused by acute SMV thrombosis, who underwent PPMT between 2011 and 2018, was reviewed. Venous mesenteric ischemia was diagnosed in the presence of SMV thrombosis, associated with the evidence of circumferential wall thickening and congestion of the jejunum loop without signs of pneumatosis or pneumoperitoneum at CT. All patients presented with mild to moderate clinical signs of peritonism, which allowed the surgery to be postponed. Initial treatment consisted of systemic heparinization through intravenous administration of unfractionated heparin adjusted to keep aPTT two times control. Treatment by PPMT was indicated in those cases where the intensity of abdominal pain increased, despite having received promptly initiated medical therapy. Informed consent was obtained from all patients. Contraindications to thrombolysis consisted of previous strokes, brain malignancies, coagulopathy, recent gastrointestinal hemorrhage, or active bleeding diathesis.

### Thrombectomy and Thrombolysis Technique

The portal vein was accessed through a percutaneous US-guided transhepatic approach, using a 21-G Chiba needle and an Accustick system (Boston Scientific, Natick, MA, USA) or through the creation of a TIPS with a Viatorr stent-graft (Gore, Flagstaff, AZ, USA). Regardless of the approach used, after accessing the portal system, a guide wire was advanced distally and a 10-Fr Aspirex device was put into place. Aspirex is a wall-contact thrombus aspiration system that works through a suction action produced by a slit situated in the distal tip of the catheter, combined with the fragmentation of the aspired clot through a rotating stainless steel spiral located in the catheter lumen. After execution of mechanical thrombectomy in the main trunk of SMV and in the portal vein when needed, occluded SMV side branches were treated via selective aspiration thrombectomy through a 7-Fr guide catheter connected to a Penumbra pump (Alameda, California, USA). At this point, a venogram was obtained, and when a partial recanalization of the SMV was observed, a side-hole infusion catheter was put into place to perform local thrombolysis for the following 18–24 h. If thrombosis persisted without signs of recanalization, a new session of mechanical thrombectomy with Aspirex was immediately repeated. Urokinase was the thrombolytic agent of choice, at the dosage of 80,000–100,000 IU per hour, based on the patient’s weight. Following the mechanical thrombectomy session, for the entire duration of the thrombolysis infusion, patients were transferred to the intensive care unit; blood coagulation and fibrinogen were strictly monitored and UK dosage adjusted consequently. Venographic evaluation was performed the following day, and a thrombectomy session was repeated if a significant amount of residual thrombus within the SMV was still present at the control. Transcatheter thrombolysis was interrupted when a patency of at least 50% of the SMV was observed with restoration of an antegrade flow to the portal system. Tract embolization was performed in all cases of transhepatic approach using Onyx 34.

### Endpoints and Follow-Up

Clinical success was defined as the resolution of symptoms. Technical success was defined as > 50% patency of the previously thrombosed vessel, with restoration of antegrade filling of SMV branches confirmed by venography at the conclusion of the treatment, and resolution of bowel wall thickening at CT.

After the procedure, all patients were discharged on lifelong oral anticoagulation therapy (INR 2.5–3.5). Follow-ups were performed using CT at the moment of discharge, after 1 month, and then 1 year after treatment. Color Doppler ultrasound follow-ups were performed at 3 and 6 months after treatment, and subsequently yearly.

## Results

The study population consisted of eight males, aged 37–81 (mean 56.5 years). All patients complained of severe abdominal pain; two patients were also suffering from nausea and three from abdominal distension. CT was repeated after completion of the treatment in order to assess bowel wall status and confirm venous patency. Etiology and causes of thrombosis were investigated in all patients (see Table 1). There were no contraindications to thrombolysis. Urokinase infusions ranged from a minimum period of 48 h to a maximum of 72 h, with dosages ranging from 3,840,000 to 5,760,000 IU. Acute SMV thrombosis was associated with portal vein thrombosis in seven out of eight cases. Different grades of portal thrombosis involvement were observed: in six cases, both the main portal trunk and the intrahepatic portal branches were affected, while in one case the right portal branch and the main portal trunk were involved. In all the cases of extensive portomesenteric thrombosis, a cavernomatous transformation was already present at the initial CT. The splenic vein was also affected in three out of seven patients, with CT findings of asymptomatic partial splenic infarction in one case.

**Table 1:** Patients demographics, clinical presentation, etiology, and comorbidities

Pt. no.	Age (years)	Sex	Symptoms	Etiology	Comorbidities
1	61	M	Abdominal pain	Antiphospholipid antibody syndrome	Chronic hepatopathy Crohn’s disease
2	69	M	Abdominal pain	Unknown	Chronic hepatopathy
3	81	M	Abdominal pain	Unknown	Chronic hepatopathy
4	55	M	Abdominal pain Nausea	Unknown	None
5	44	M	Abdominal pain	Unknown	Crohn’s disease
6	37	M	Abdominal pain	Unknown	Chronic hepatopathy
7	59	M	Abdominal pain	Unknown	Cirrhosis
8	46	M	Abdominal pain	Unknown	Myeloproliferative disease

In five out of eight patients, a right portal branch was punctured using a transjugular (TJ) approach and a portosystemic shunt (TIPS) was created in order to access the SMV and perform thrombectomy. In three out of five cases, a TIPS was created by targeting a landing site on a thrombosed portal branch because of a coexisting intra- and extrahepatic portal vein thrombosis. In the remaining three patients, a transhepatic ultrasound-guided approach was preferred. Clinical and technical success was obtained in all cases and coincided with flow restoration in the SMV. SMV remained patent at all follow-up controls in all cases. There were two cases of early portal rethrombosis with the evidence of cavernomatosis at the end of the treatment, despite successful intraoperative recanalization after PPMT. In these cases, the final venography showed good outflow from the restored SMV to the intrahepatic portal branches, or to the TIPS via the cavernomatosis. Portal vein thrombosis reoccurred postoperatively in three cases, at 1-month FU. TIPS patency was confirmed at dismissal, but during follow-up, shunt obstruction was detected in three out of four cases, respectively, at 1- , 6- and 12-month FU, despite anticoagulation therapy. In these patients, SMV was patent at every FU control and flow to the portal intrahepatic branches was ensured via the cavernomatosis. Neither explorative nor resective surgery was required after PPMT; mean hospitalization stay was 19.4 days (9–38). Mean follow-up (in remaining seven patients) after discharge was 37.7 months (12–84 months).

### Complications

One patient developed an abdominal wall hematoma after 48 h of local thrombolysis and underwent CT, which revealed the presence of active bleeding fed from the superior left epigastric artery. Thrombolysis was suspended, and the bleeding was successfully treated via a transfemoral catheterization and a superselective embolization with Onyx.

One patient died of multi-organ failure (MOF) 35 days after treatment. This patient had initially undergone surgery because of bowel necrosis present at the onset with severe peritonism, sustained by portomesenteric thrombosis. However, 3 days after resection, there was a recurrence of mesenteric ischemia due to a distal extension of the SMV thrombosis. In order to avoid surgical reintervention, PPMT was performed via a TIPS creation because of the presence of an extensive portomesenteric thrombosis. After 48 h of thrombolysis, complete symptom resolution was obtained. After 6 days of well-being, the patient developed a fever caused by fluid collection. A drainage was put into place, and the patient was put on antibiotic therapy. After 18 days, despite resolution of the collection, MOF occurred and the patient died (Table 2).

**Table 2:** Treatment modalities and outcome

Thrombosis site	Route	Treatment	Thrombolytic agent	Thrombolysis duration/dose (IU)	Post-treatment cavernomatosis	Hospital stay	Complications	Clinical outcome	Follow-up
SMV, SV, PV (extensive extra and intrahepatic branches)	TJ	TIPS, PMT + TT	UK	48 h/4,800,000	At 1 month	12	None	Symptom Resolution	2 years
SMV, SV, PV(extensive extra and intrahepatic branches)	TH	PMT + TT	UK	48 h/4,800,000	At discharge	16	None	Symptom Resolution	1 year
SMV, PV (extra and intrahepatic right portal branch)	TH	PMT + TT	UK	48 h/3,840,000	No	9	Bleeding	Symptom Resolution	1 year
SMV	TH	PMT + TT	UK	72 h/5,760,000	No	11	None	Symptom Resolution	2 years
SMV, SV, PV (extensive extra and intrahepatic branches)	TH + TJ	TIPS, PMT + TT	UK	52 h/4,160,000	At 1 month	13	None	Symptom Resolution	4 years
SMV, PV (extensive extra and intrahepatic branches)	TJ	TIPS, PMT + TT	UK	68 h/5,440,000	At discharge	16	None	Symptom Resolution	7 years
SMV, PV, SV (extensive extra and intrahepatic branches)	TJ	TIPS + TT	UK	72 h/5,760,000	No	12	None	Symptom Resolution	5 years
SMV, PV (extensive extra and intrahepatic branches)	TJ	Surgery TIPS + TT	UK	48 h/4,800,000	Yes (post-procedural)	38	Death for MOF	Resolution of pain MOF	Death at 35 days

*SMV* superior mesenteric vein, *SV* splenic vein, *PV* portal vein, *UK* urokinase, *TIPS* transjugular intrahepatic portosystemic shunt, *PMT* percutaneous mechanical thrombectomy, *TH* transhepatic, *TJ* transjugular, *MOF* multi-organ failure, *TT* transcatheter thrombolysis

## Discussion

Although to date there is no consensus on treatment of acute SMV thrombosis, once it has been diagnosed, the primary goal of therapy must be to avoid the process leading to transmural infarction, perforation, and severe peritonitis. Systemic heparinization may improve recanalization rates up to 80% [7] and can be beneficial in terms of patient survival [8]. However, anticoagulation alone is associated with recurrence of thrombotic events in 3–40% of cases [9].

Surgery is mandatory in the presence of severe peritonitis or perforation [5], but even when the diagnosis is established promptly, 30-day mortality rates in acute SMV thrombosis range from 13 to 50% with traditional treatment of anticoagulation and bowel resection [10]. However, endovascular treatment by means of transcatheter thrombolysis alone may require high dosages and long infusion times, with an increase of up to 60% in the risk of bleeding and intracranial or gastrointestinal hemorrhage [11, 12]. Thrombectomy by manual aspiration or by devices which mechanically debulk and aspire the thrombus has been used in the last years and has demonstrated encouraging results [13]. In particular, percutaneous mechanical thrombectomy has proven to be effective in reducing the dosage and the infusion time of thrombolytics [14, 15].

Reviewing the PubMed database, 30 reported cases of patients with acute SMV thrombosis who had undergone percutaneous mechanical thrombectomy were found [16–26] (Table 3). In the 93.3% of cases, the procedure was technically successful, flow in the SMV was restored, and abdominal symptoms resolved.

**Table 3:** Previously published studies

Author (year)	Patients	Thrombectomy device	Route	Thrombolytic agent	Dose–duration	Clinical outcome	Surgical resection	Complications
Rosen, (2000)	1	Angiojet	TH + TA	–	–	Successful	No	–
Lopera (2002)	2	Oasis, arrow-Trerotola	TH	Urokinase	12 h–100,000 IU/h	Successful	No	–
Kim (2005)	7	Angiojet, Amplatz	TH	rt-PA Urokinase	Up to 45 h Up to 8.5 million IU	6 Successful 1 unsuccessful	No	1 Hemothorax 1 death
Takahashi (2005)	1	Oasis	TH	Urokinase	72 h–240,000 IU/day	Successful	Yes	–
Zhou (2007)	2	Angiojet	TH	rt-PA	12 h	Successful	No	–
Wasselius (2014)	1	Angiojet	TJ + TIPS + TA	rt-PA	33 h	Successful	No	–
Jun (2014)	2	Angiojet	TH	Urokinase	48 h–100,000 IU/h	Successful	No	–
Lorenz (2014)	1	Trellis	TJ	rt-PA	12 h	Successful	No	1 Bleeding requiring transfusions
Song (2017)	8	Angiojet	TH	Urokinase	24–48 h to 500,000 IU/day	Successful	No	1 Bleeding requiring transfusions
Syed (2018)	2	Angiojet	TH	rt-PA	–	Successful	No	–
Kuetting (2018)	3	Angiojet	TIPS	Urokinase	22–52 h to 100,000 IU/h	Successful in 2/3 cases	No	2 Hematuria

*UK* urokinase, *rt*-*PA* recombinant tissue plasminogen activator, *TA* transarterial, *TH* transhepatic, *TJ* transjugular

Percutaneous mechanical thrombectomy, either alone or followed by local thrombolysis, was performed in those series using a TJ or a TH approach, in some cases combined with indirect transarterial thrombolytics infusion through the superior mesenteric artery. The transarterial approach is the least efficient because the thrombolytics are dispersed through the patent arterial branches, without direct vehiculation into the thrombosed vessels. The TIPS approach is invasive and technically challenging, especially in portal vein thrombosis, and it may cause dispersion of the drug into the systemic venous circle. The rationale of this approach is to create a low pressure system which provides a valid outflow for the recanalized vessels in the case of complete extensive thrombosis of the portomesenteric system. A transhepatic ultrasound-guided approach is less invasive, quicker to achieve for operators, even in the case of puncture of a thrombosed branch, and allows direct, maximized thrombolytic action within the thrombosed vessels. Nevertheless, it requires tract embolization at the end of the treatment, since a large diameter introducer is used (9–11 Fr). It was the first choice in this series for non-cirrhotic patients with patent portal branches representing a potential outflow for the recanalized SMV. In this study, tract embolization was performed using Onyx 34, which was chosen over coils because it allows hemostasis even during ongoing heparinization or thrombolytic therapy.

Clinical success was obtained in this series in seven out of eight patients, for whom SMV patency was confirmed in FU controls, despite four cases of portal thrombosis recurrence with cavernomatous transformation. It is not clear why portal vein thrombosis recurred, whereas SMV thrombosis did not. A portal cavernoma takes from 6 to 20 days to form after acute thrombosis [27]. In six out of the eight cases, a preexisting cavernomatosis was already present at the moment of PPMT, indicating a potential subacute portal vein thrombosis, which could explain the unusual high rate of rethrombosis; moreover, intravascular ultrasound (IVUS) was not used in this study. Although there is limited evidence of the use of IVUS in the management of portomesenteric thrombosis [28], it has been shown to improve patency outcomes in the iliofemoral DVTs [29]. Therefore, investigations should be carried out as to whether the use of IVUS could also be beneficial in improving patency rates after portomesenteric thrombectomy and thrombolysis. Although cavernomatous transformation is usually considered to be a technical failure of thrombolysis or of mechanical thrombectomy of the portal vein, it was not associated in this study with clinical failure because the cavernoma provided a functional outflow from the recanalized SMV into the intrahepatic circle.

A single case of major bleeding in this series was observed, which was treated with embolization without clinical consequences. No other major adverse events were observed. Intestinal surgical resection after endovascular treatment was not necessary in any of the cases, while Yang [30] in 2015 had described a 50% resection rate following transcatheter thrombolysis via a combined TJ and SMA approach.

All patients were clinically stable at discharge and have returned to their daily life activities. They are all being treated with anticoagulation therapy and those with portal cavernomatosis continue to be monitored for portal hypertension complications (Figs. 1, 2, 3, 4, 5, 6, 7, 8, 9).

**Fig. 1A, B, 2:**
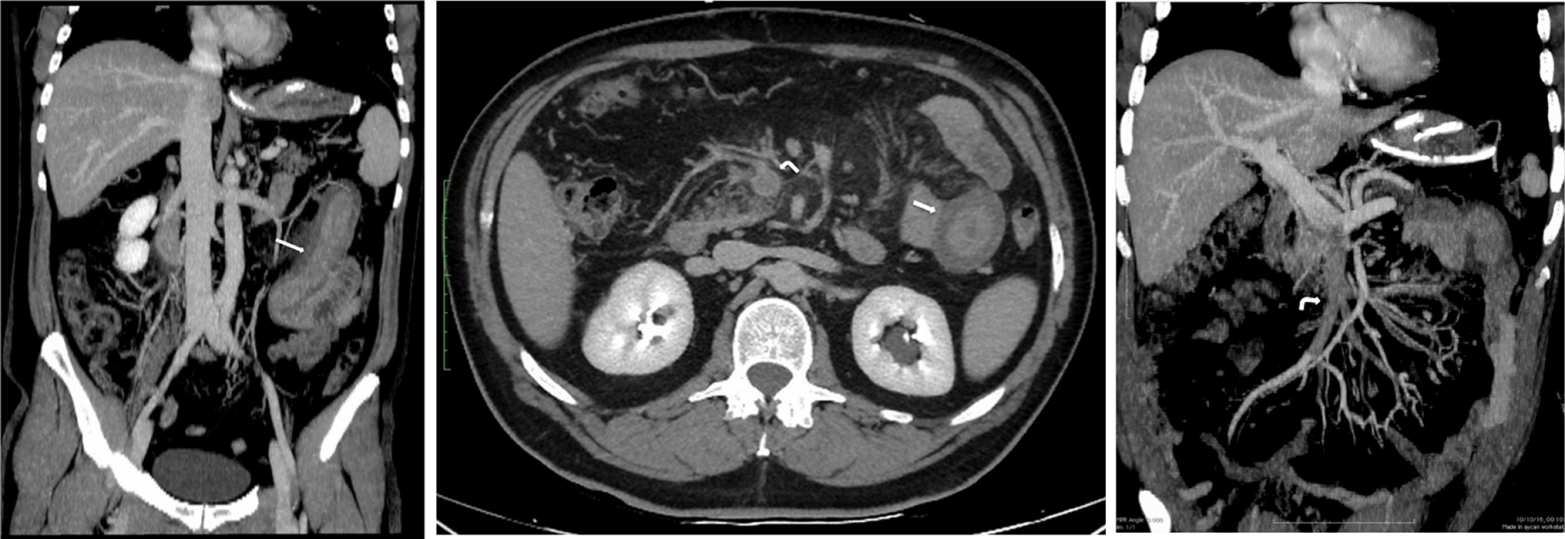
Preoperative contrast-enhanced CT scans of a 55-year-old male (patient No. 4) show thickening of jejunum loop (arrow) and extensive thrombosis of the SMV (curved arrow)

**Fig. 3:**
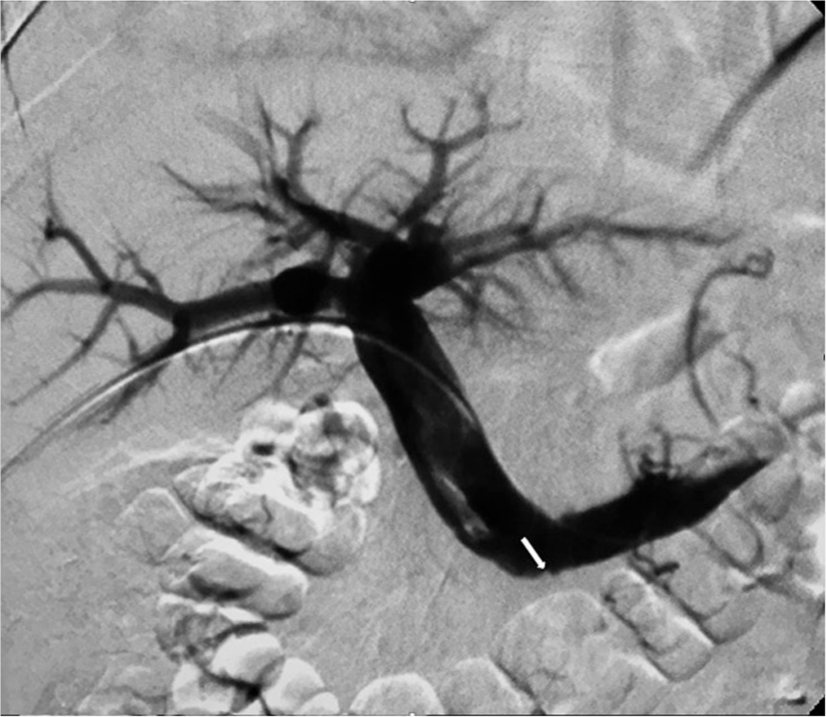
Transhepatic venography (anteroposterior view) shows thrombosis of the proximal superior mesenteric vein (arrow) and patency of portal vein

**Fig. 4:**
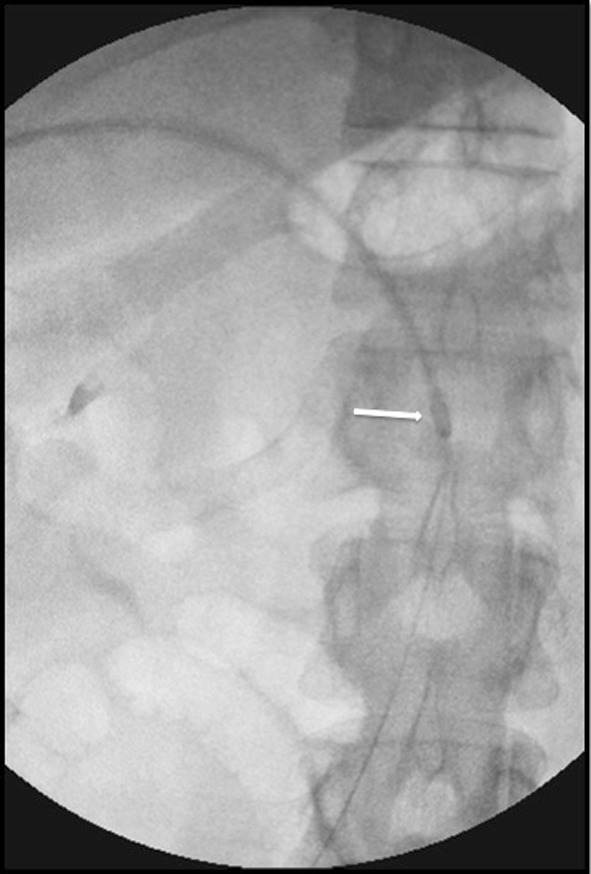
Advancement of the 10-Fr Aspirex catheter (arrow) into the thrombosed SMV under fluoroscopic guidance

**Fig. 5:**
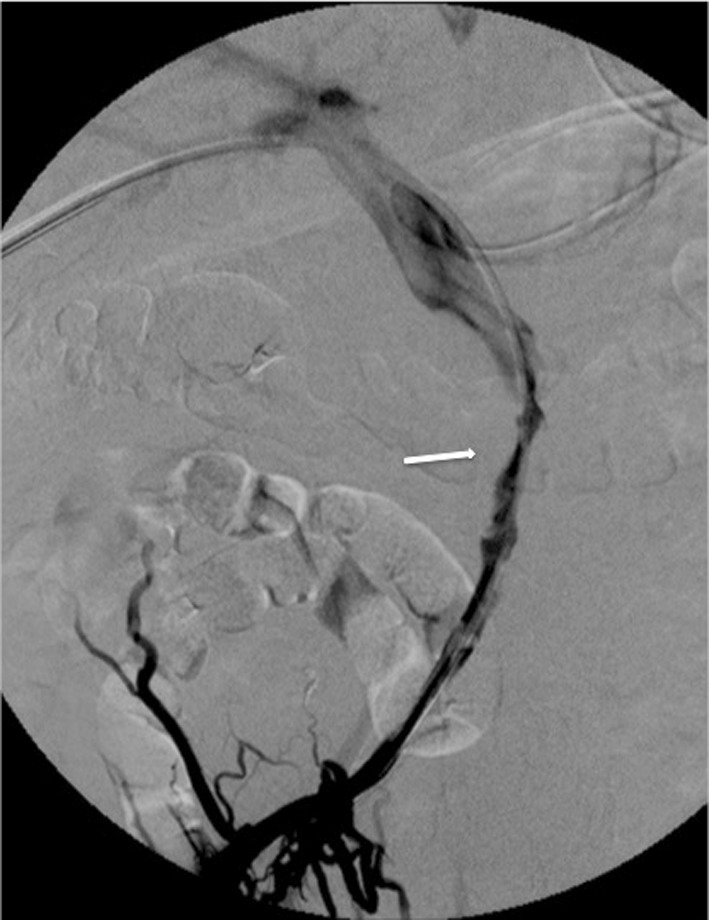
Control venography immediately after mechanical thrombectomy with Aspirex and before local transcatheter thrombolysis shows partial patency of the SMV with antegrade flow; a narrowing of the main trunk of the vessel caused by residual thrombosis is still evident (arrow)

**Fig. 6:**
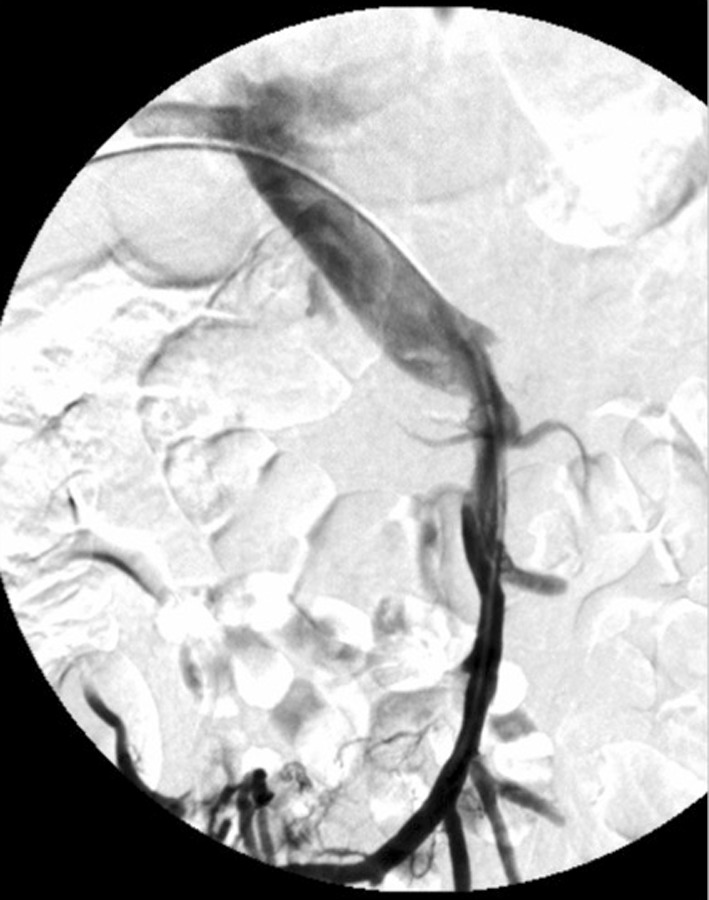
Control venogram after 48 h of local thrombolysis demonstrates a significant improvement of flow within the recanalized SMV

**Fig. 7:**
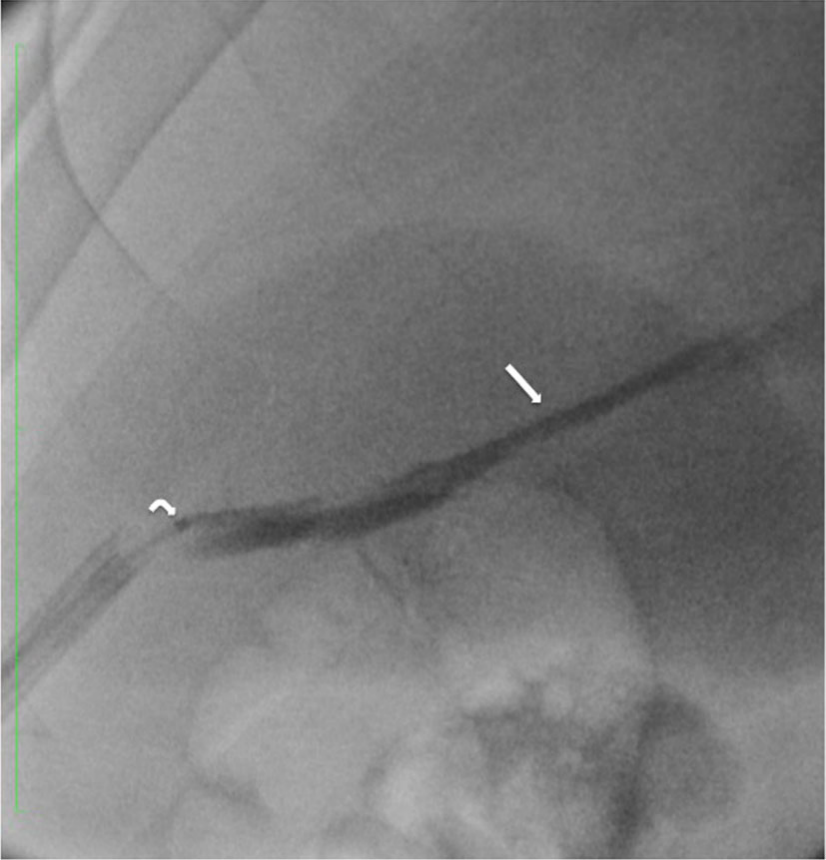
Tract embolization with Onyx 34: the 11-Fr introducer is partially withdrawn while the radiopaque embolic agent (arrow) is injected through a 2.7 DMSO-compatible microcatheter (curved arrow)

**Fig. 8:**
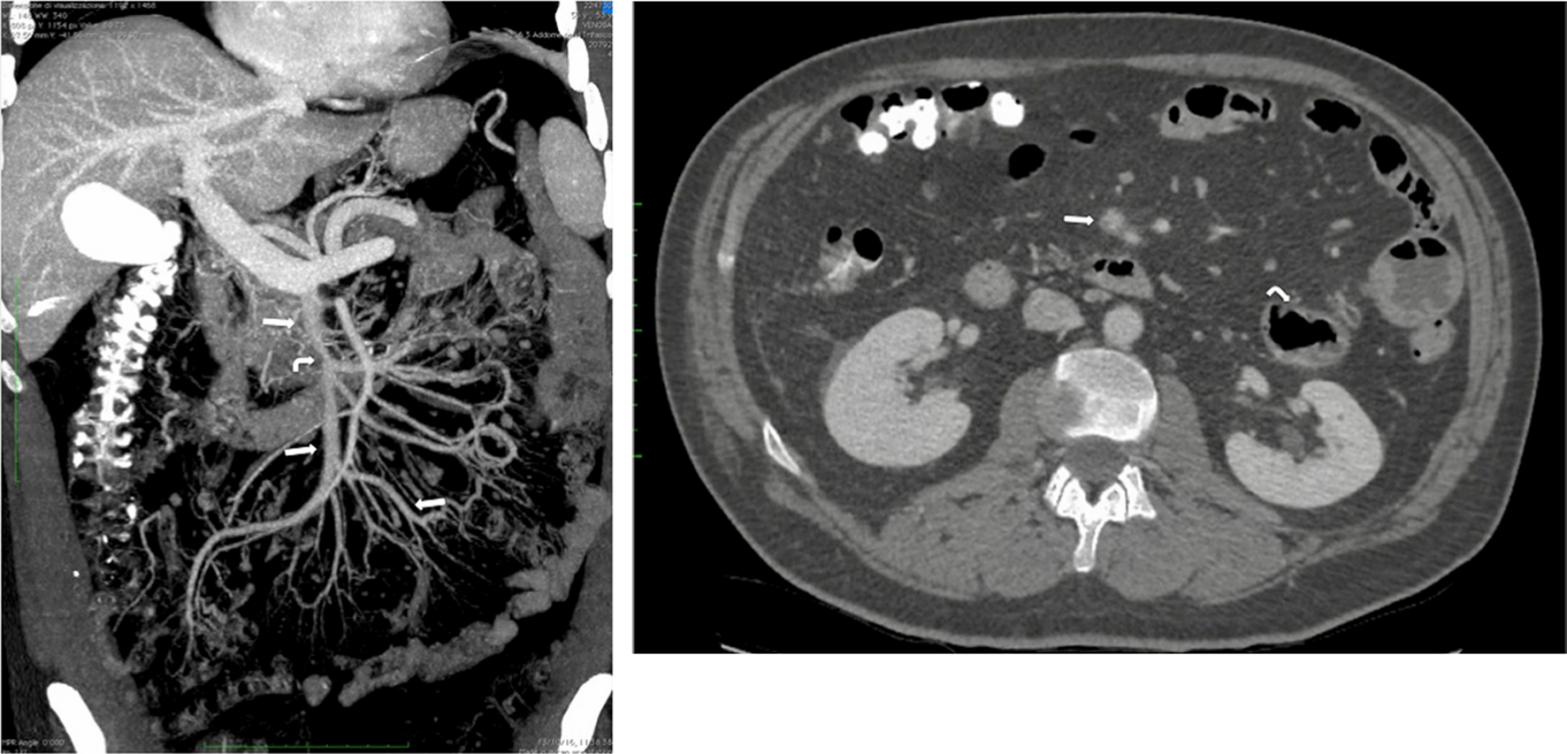
**a** Pre-discharge postoperative CT confirms recanalization of both main trunk and side branches of the SMV (arrow); a residual thrombosis is still evident into the SMV lumen (curved arrow). **b** Pre-discharge postoperative CT shows resolution of jejunal thickening (curved arrows) and recanalization of the SMV main trunk (arrow)

**Fig. 9:**
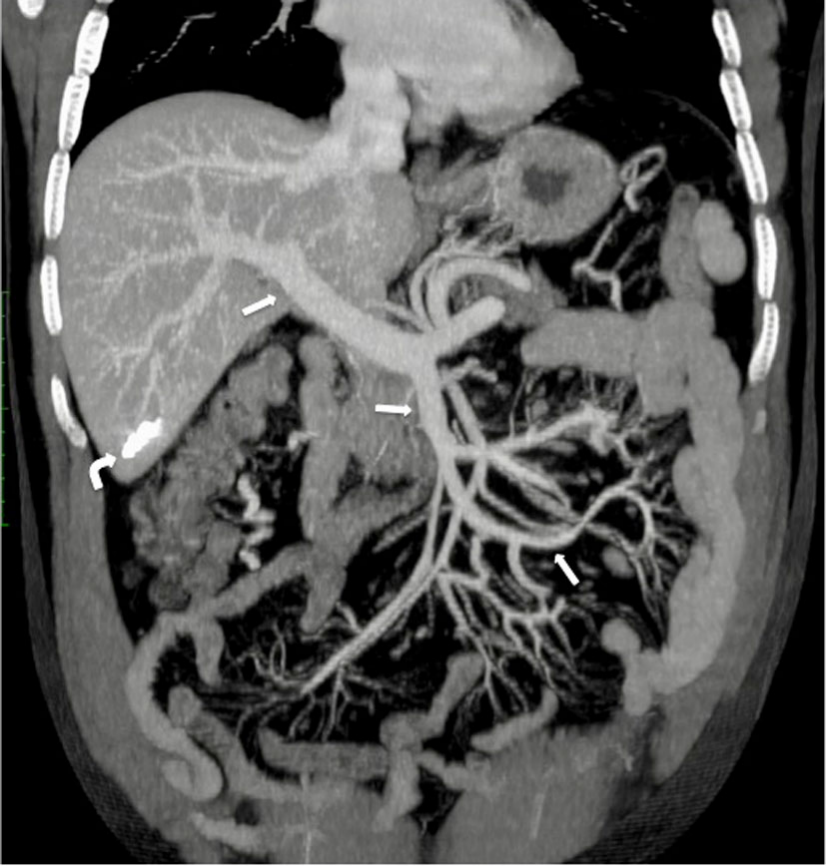
One-year follow-up CT shows complete recanalization of the SMV and PV (arrow); the hyperdense embolized hepatic tract is still visible within the sixth hepatic segment (curved arrow)

## Conclusion

At present, there is no agreement on the optimal treatment strategy for acute mesenteric venous ischemia, because of the rarity of the condition and the lack of RCTs. In fact, all available data come from single-center small series or case reports. However, results from those series would seem to encourage an early endovascular approach, particularly with percutaneous pharmaco-mechanical thrombectomy, in order to achieve early mesenteric revascularization and avoid surgical resection. According to the findings in this study, treatment of acute SMV thrombosis with PPMT in patients without symptoms and signs of surgical abdomen seemed safe and effective, with a 87.5% long-term survival rate and a 12.5% major complication rate.
